# Supplementary material for: Identification and Characterization of Novel Founder Mutations in NDRG1: Refining the Genetic Landscape of Charcot–Marie–Tooth Disease Type 4D in Bulgaria
Source: Int J Mol Sci. 2024 Aug 21;25(16):9047. doi: 10.3390/ijms25169047 (PMC11354586; doi:10.3390/ijms25169047)
Supplement: Supplementary file 1 [file ijms-25-09047-s001.zip › TableS1 - primers.pdf]

Table S1: Primers used in this study.

| Label | Sequence | Use |
|---|---|---|
| D8S1011_F_M13 | 5'-AGCGGATAACAATTTCACACAGGCTGGTGACAGAGTGATAGC-3' | Haplotyping |
| D8S1011_R | 5'-GTTTCTTCAGAGCTCCCATACAAAGG-3' | Haplotyping |
| D8S1720_F_M13 | 5'-AGCGGATAACAATTTCACACAGGGTGCCACCTGCCTGAA-3' | Haplotyping |
| D8S1720_R | 5'-GTTTCTTCCACTACCTATTTAGAGAGGCCA-3' | Haplotyping |
| D8S1712_F_M13 | 5'-AGCGGATAACAATTTCACACAGGAATGCACTGACTGGAGACG-3' | Haplotyping |
| D8S1712_R | 5'-GTTTCTTACTCACAGATTGTTGCGAC-3' | Haplotyping |
| D8S1765_F_M13 | 5'-AGCGGATAACAATTTCACACAGGGATCGCACCATTGCACTC-3' | Haplotyping |
| D8S1765_R | 5'-GTTTCTTGGGCCTTACTTTTAGCCTTTAAC-3' | Haplotyping |
| D8S557_F_M13 | 5'-AGCGGATAACAATTTCACACAGGCAGGGTACAGACATGCTTG-3' | Haplotyping |
| D8S557_R | 5'-GTTTCTTCCTGGGGTCCTAGAGATTT-3' | Haplotyping |
| D8S1835_F_M13 | 5'-AGCGGATAACAATTTCACACAGGGGAAACTCCCCATAGCCAT-3' | Haplotyping |
| D8S1835_R | 5'-GTTTCTTCCCAGGATTCCATGTGTG-3' | Haplotyping |
| D8S558_F_M13 | 5'-AGCGGATAACAATTTCACACAGGGGAACCACGCTTCGTTT-3' | Haplotyping |
| D8S558_R | 5'-GTTTCTTGGGGCTTTAAGACCCAT-3' | Haplotyping |
| D8S529_F_M13 | 5'-AGCGGATAACAATTTCACACAGGTGCCCCTAACCTGTCT-3' | Haplotyping |
| D8S529_R | 5'-GTTTCTTTTAGCCTCCAATATGTCTCT-3' | Haplotyping |
| D8S256_F_M13 | 5'-AGCGGATAACAATTTCACACAGGGTTCAAGGGCTCAGGGTTCT-3' | Haplotyping |
| D8S256_R | 5'-GTTTCTTCTTCCACCTTTAGCCAAGGA-3' | Haplotyping |
| D8S1708_F_M13 | 5'-AGCGGATAACAATTTCACACAGGAGTGGCTTAGGACGTGC-3' | Haplotyping |
| D8S1708_R | 5'-GTTTCTTCAGGTGAACTGCTGTGTTTA-3' | Haplotyping |
| D8S1746_F_M13 | 5'-AGCGGATAACAATTTCACACAGGGCTTAGACTATGGGACCACAC-3' | Haplotyping |
| D8S1746_R | 5'-GTTTCTTCAGTGAACATCAACCCTGC-3' | Haplotyping |
| D8S554_F_M13 | 5'-AGCGGATAACAATTTCACACAGGTTTCCAGACAGGGCCTA-3' | Haplotyping |
| D8S554_R | 5'-GTTTCTTAATGCACAGGACATCCTTT-3' | Haplotyping |
| D8S534_F_M13 | 5'-AGCGGATAACAATTTCACACAGGTGCAGCACGACTCACAATAA-3' | Haplotyping |
| D8S534_R | 5'-GTTTCTTAGCTTAATGTCTTCTCAGTTCATCC-3' | Haplotyping |
| M13_tag | 5'-FAM-AGCGGATAACAATTTCACACAGG-3' | Haplotyping |
| cDNA_NDRG1_ex6F | 5'-AGGACATGCAGGAGATCACC-3' | cDNA analysis |

| | | |
|---|---|---|
| cDNA_NDRG1_ex6R | 5'-AGTTAGGATGTAGGCGCCTG-3' | cDNA analysis |
| Ex6F-FAM | 5'-FAM-AGGACATGCAGGAGATCACC-3' | Allele quantification |
| Ex6_R | 5'-AGTTAGGATGTAGGCGCCTG-3' | Allele quantification |
| cDNA_NDRG1_ex9F | 5'-CTGGCTGAAATGCTTCCTGG-3' | cDNA analysis |
| cDNA_NDRG1_ex9R | 5'-TGATGAACAGGTGCAGGTTG-3' | cDNA analysis |
| Ex9F-FAM | 5'-FAM-CTGGCTGAAATGCTTCCTGG-3' | Allele quantification |
| Ex9_R | 5'-TGATGAACAGGTGCAGGTTG-3' | Allele quantification |
