# Supplementary figures and images for: Identification and Characterization of Novel Founder Mutations in NDRG1: Refining the Genetic Landscape of Charcot–Marie–Tooth Disease Type 4D in Bulgaria
Source: Int J Mol Sci. 2024 Aug 21;25(16):9047. doi: 10.3390/ijms25169047 (PMC11354586; doi:10.3390/ijms25169047)

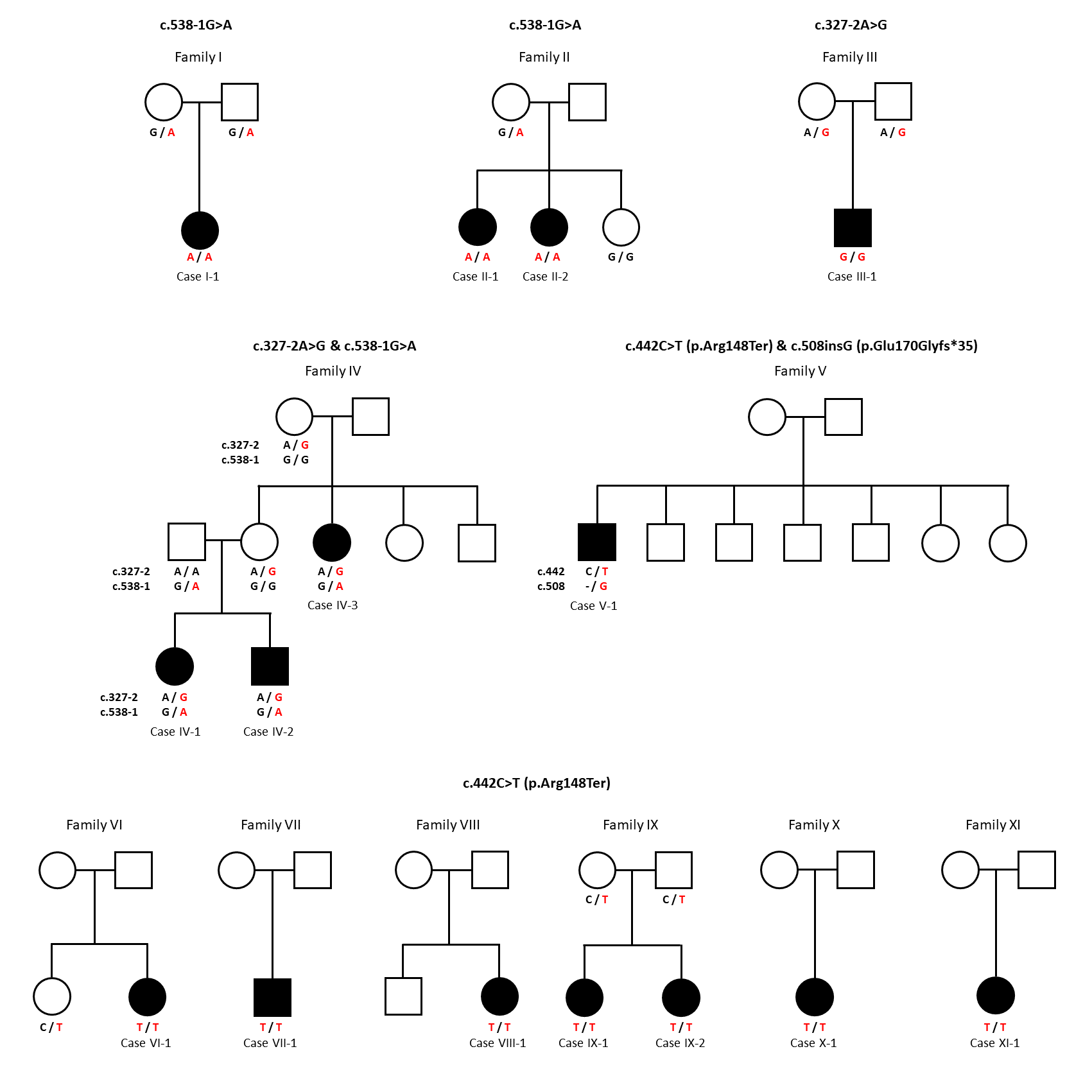

Supplement: Supplementary file 1 [file ijms-25-09047-s001.zip › FigureS1 - pedigrees.png]

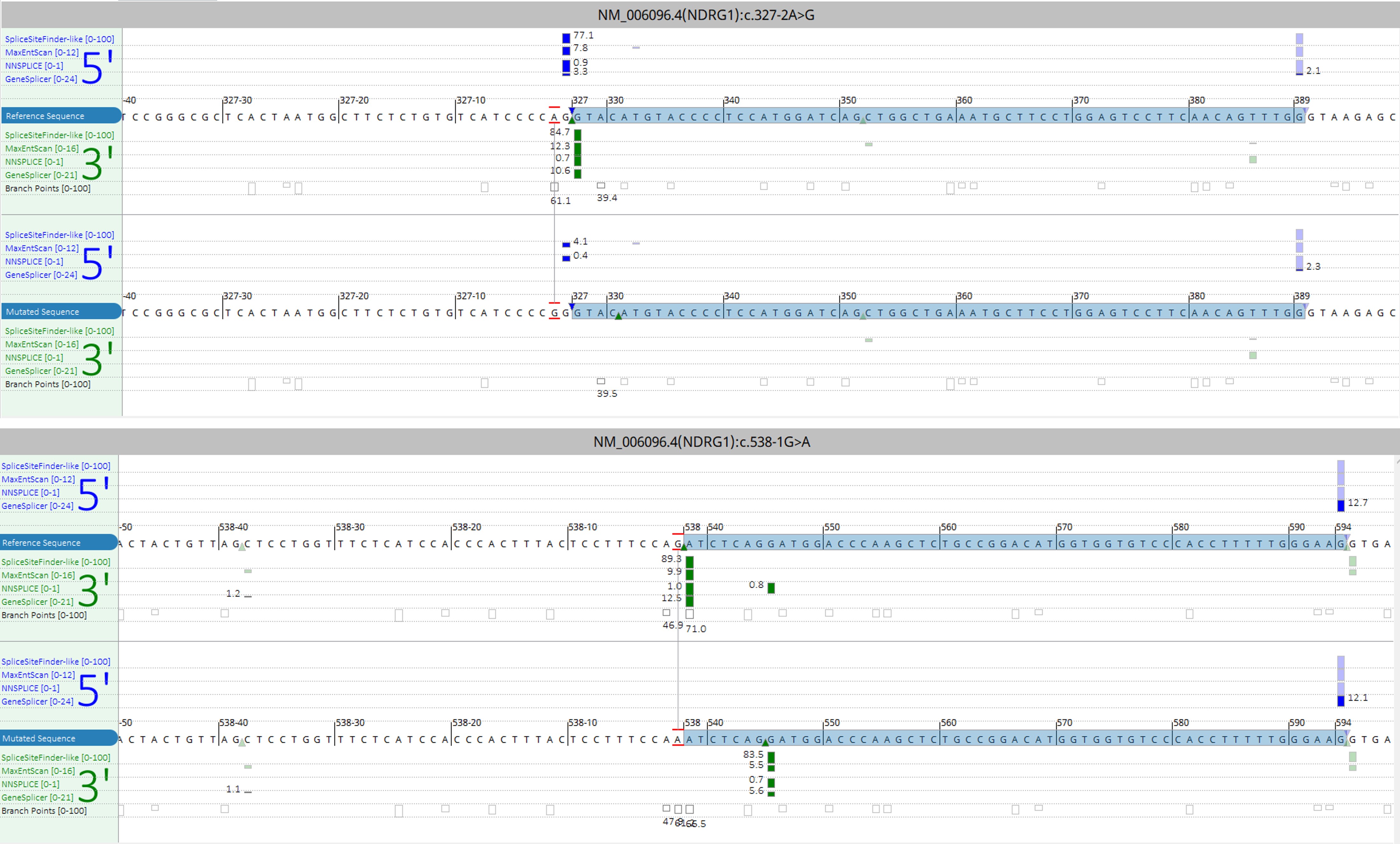

Supplement: Supplementary file 1 [file ijms-25-09047-s001.zip › FigureS2 - splicing prediction.jpg]

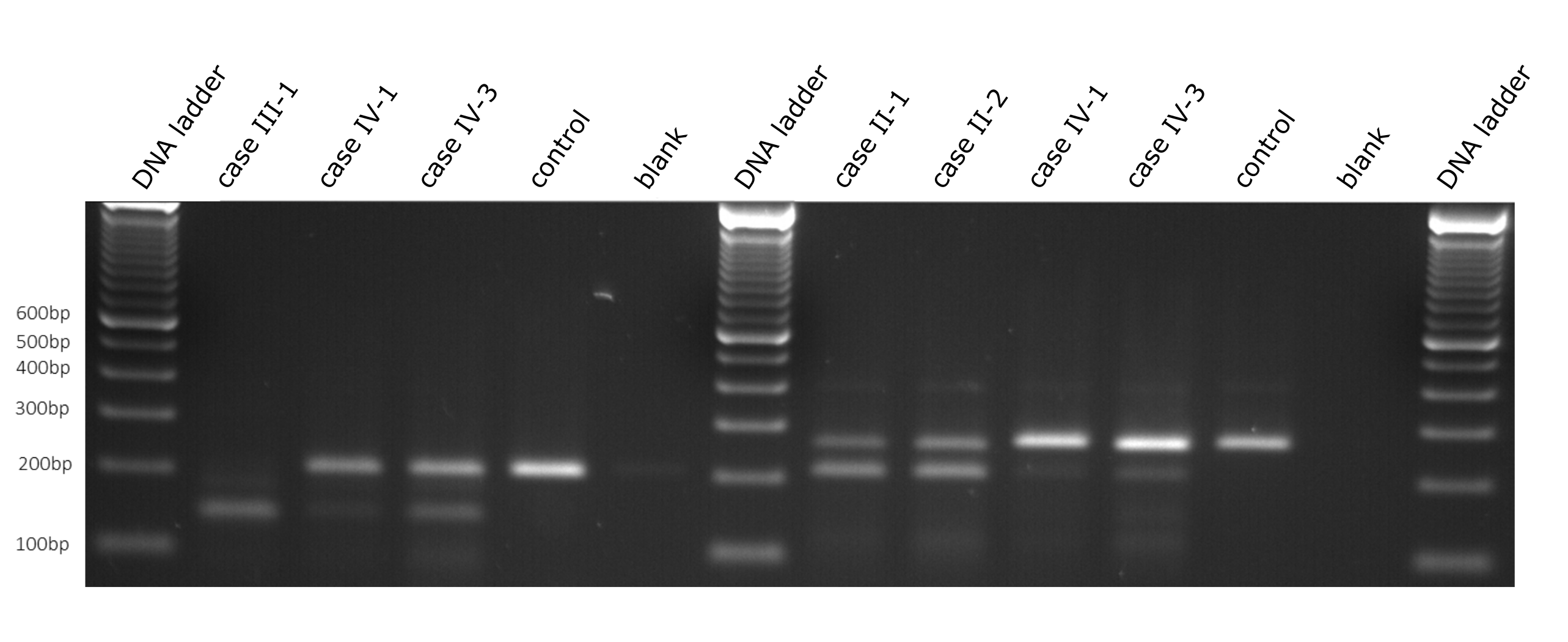

Supplement: Supplementary file 1 [file ijms-25-09047-s001.zip › FigureS3 - heteroduplex.png]

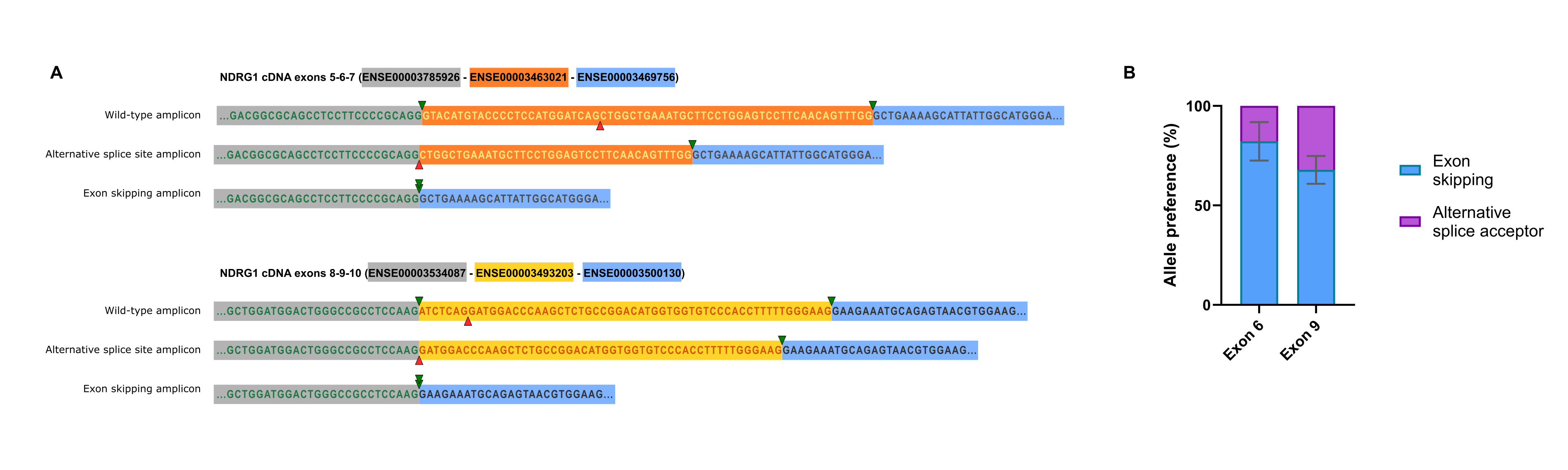

Supplement: Supplementary file 1 [file ijms-25-09047-s001.zip › FigureS4 - amplicon_details.png]
